# Physiological stress differentially impacts cognitive performance during—and memory following—simulated police encounters with persons experiencing a mental health crisis

**DOI:** 10.3389/fpsyg.2025.1549752

**Published:** 2025-03-18

**Authors:** Hannah Marlatte, Paula M. Di Nota, Judith P. Andersen

**Affiliations:** ^1^Department of Psychology, University of Toronto St. George, Toronto, ON, Canada; ^2^Rotman Research Institute, Baycrest Health Sciences, North York, ON, Canada; ^3^Department of Psychology, University of Toronto Mississauga, Mississauga, ON, Canada

**Keywords:** memory, perception, situational awareness, cognition, police, stress physiology, crisis intervention

## Abstract

Police officers frequently make decisions under stress and require accurate memories of their perceptions and actions for subsequent investigations. Recognizing that police are frequently called to assist people experiencing a mental health crisis, it is of critical importance to public safety to understand the role of stress on officers’ cognition when navigating such encounters. Despite this, how the timing of experiencing stress impacts officer cognition is understudied in applied police contexts and therefore remains unclear. To address this gap in the literature, we analyzed data from a study of 57 police officers who wore heart rate monitors to record physiological arousal before, during, and after two reality-based scenarios (i.e., simulated calls for service) with individuals experiencing mental distress. Scenarios were audio-recorded, transcribed, and coded to measure officers’ perceptual memory of important elements in each scene, procedural memory to enact best practices, post-incident memory of their own actions, and higher-level situational understanding. We found a nuanced relationship between the timing of stress and cognitive performance, such that higher heart rate before and during scenarios improved understanding, decision making, and the appropriate choice of use of force option, but at the expense of officers’ spatial processing. Increased heart rate during the post-incident debrief was associated with the following: making a lethal force error during the scenario, decreased memory for perceptual aspects of the scenario, and impaired recall of one’s own actions. Older and more experienced officers exhibited overall lower physiological arousal, and female officers demonstrated better cognitive performance compared to male officers. These results have practical implications in operational, training, evaluation, and testimonial police contexts and can inform future interventions aimed to improve outcomes when navigating stressful encounters, including crisis intervention.

## Introduction

Police frequently navigate stressful encounters and make high-risk decisions with limited information, sometimes under significant time pressure (Baldwin et al., 2022). The potentially severe personal, societal, legal, and public safety consequences of officers’ decision-making may further exacerbate the impact of stress on performance. Furthermore, officers’ memory of the complex context and rationale underlying their decisions needs to be preserved for investigative or testimonial purposes for years to come. Accordingly, police are expected to show a high caliber of cognitive performance, including accurate perception, understanding, and recall of work-related encounters, as well as enhanced procedural memory for complex motor skills acquired through training and experience (Jones et al., 2018). This may be especially relevant when navigating situations with individuals in mental health crisis, which may be less predictable. Despite the importance of understanding how the timing of stress responses may impact cognitive performance in applied police contexts, little research has examined the nuanced interplay between these factors (Di Nota and Huhta, 2019; Di Nota et al., 2020). To address this gap in the literature, the present study aims to assess how physiological arousal before, during, and after simulated critical incident scenarios involving individuals in a mental health crisis impacts cognitive performance in occupationally relevant police settings.

Police are often the first to respond to persons in mental health crisis. They are therefore expected to frequently navigate situations involving individuals presenting either suicidal and/or violent behaviors that could be dangerous to themselves or others (Huey et al., 2021; Livingston, 2016). These calls can be especially challenging for officers, given the greater amount of complexity and resources they require (Godfredson et al., 2011; Charette et al., 2014). Further, the limited amount of training provided to officers is unlikely to cover the variety of situations that can transpire (Wood et al., 2021; Pelfrey and Young, 2020). There are additional factors that may exacerbate stress for both the individual and the officer, including the increased likelihood of UOF in such calls and the perceptions of police held by individuals experiencing mental illness (Huey et al., 2021; Fyfe, 2000; Watson et al., 2008a). The fact that upwards of 10% of police calls for service involve interacting with persons in crisis only underscores the need for research on police cognition during such encounters (Fry et al., 2002; Godfredson et al., 2011).

A substantial amount of applied research has examined perceptual, motor, and cognitive processing in stressful police contexts more broadly. The literature highlights deficits associated with stress, such as: fine, gross (i.e., arrest, self-defence skills), and visuo-motor behaviors (i.e., gaze, fixation, blinking); perception of time, distance, and positioning, and; critical decision-making related to UOF (for reviews, see Anderson et al., 2019; Di Nota and Huhta, 2019). Increased physiological arousal and accompanying performance decrements are observed during high-threat reality-based scenarios commonly used in operational field settings, as well as law enforcement training, assessment, and research (Anderson et al., 2002; Baldwin et al., 2019; Baldwin et al., 2022; Nieuwenhuys and Oudejans, 2011; Nieuwenhuys et al., 2012a, 2012b). Reality-based scenarios serve an important function by affording officers an opportunity to safely practice relevant skills in dynamic and representative environments (e.g., utilizing actors, simulated injuries and weapons, or varying levels of threat or complexity) including rare occurrences like mass casualty events or lethal force encounters (Baldwin et al., 2018; Jenkins et al., 2020; Körner and Staller, 2021).

Deliberate skill practice through reality-based training develops expertise, characterized by increased accuracy, speed and flexibility of performance (Ericsson, 2008). By detecting patterns of information acquired through training and experience, expert decision-making strategies are increasingly intuitive, automatic, and elicit habitual responses that are less susceptible to interference from stress (Klein et al., 1989; Kahneman, 2003; Vickers and Lewinski, 2012; Arble et al., 2019). Effective decision-making is fundamentally dependent on situational awareness (SA), another essential cognitive skill among police. SA can be defined as three components: (1) perception to the surrounding environment, (2) understanding or ‘sensemaking’ based on current and prior knowledge, and (3) prediction of what may happen next (Endsley, 1995; Di Nota and Huhta, 2019). Recent reconceptualizations of SA in police contexts have also included awareness of oneself and how they impact the unfolding situation as an additional component of SA (“self-awareness”; Huhta et al., 2022, 2023). Despite these advances, it remains unclear how stress physiology impacts SA and subsequent memory of situational information during encounters with persons in crisis.

Basic science research conceptualizes that our episodic memory of a situation depends on multiple cognitive processes, including perception, attention, spatial processing, and higher-level cognitive control and inference. It is often considered a constructive process that shapes both present perception and later recall (Bartlett, 1932; Schacter, 2012b). Our experience of an event is first filtered through one’s existing knowledge, relevant experiences, and current goals, which influence what is perceived, attended to, and later used for memory recollection (Gilboa and Marlatte, 2017; Xue, 2018). Our recollection of the event is influenced by how the memory is probed, including any new incoming information, as well as our current attitudes, beliefs, and motivations (Ozubko, 2011; Loftus, 2005; Naveh-Benjamin and Kilb, 2012; Bartlett, 1932; Schacter, 2012a). Our memory for events is therefore not limited to the original experience, but rather a dynamic process that can be influenced by a variety of factors. This constructive nature is adaptive as it increases the efficient functioning of perception, understanding, and knowledge organization, however, can also lead to cognitive errors such as false memories (Schacter et al., 1998).

Stress can also profoundly impact episodic memory formation and recall. However, the impact of stress depends on what stage of memory processing the stressor occurs in (Shields et al., 2017): experiencing stress during retrieval broadly impairs memory, whereas experiencing stress before encoding may impair memory unless the encoding period was short, or the learned materials were relevant to the stressor. Although there is limited research on the effect the timing of stress has on aspects of memory within police contexts, stress-induced perceptual distortions are commonly reported (Baldwin et al., 2022; Klinger and Brunson, 2009). Such distortions include experiencing tunnel vision, behaving on ‘autopilot’, and distorted time and sound perception, which can impact what officers encode and in turn lead to false memories (Hope et al., 2016). Prior studies mainly investigate the final recall stage of police memory, whereas systematic investigation of how stress influences encoding remains underexplored (Di Nota et al., 2020).

The present study examines how the timing of stress influences police officers’ cognitive performance during simulated encounters involving individuals in mental health crises. By using coded and transcribed audio recordings of verifiable events, we can examine the impact of stress on both in-the-moment performance and subsequent recall controlling for idiosyncratic differences in memory construction. Stress is quantified through physiological arousal (i.e., maximum heart rate, HRMax) relative to the individual’s resting heart rate. HRMax was measured during (1) pre-scenario briefing, (2) the scenario itself, and (3) post-scenario debriefing. Cognitive performance is defined as perception, understanding, decision-making, and memory of the simulated encounters. We hypothesize that pre-scenario HRMax and HRMax during the scenarios will be related to aspects of cognitive performance, but with no clear direction of effect given the lack of existing literature in this applied setting. Measures of cognitive performance may be differentially impacted by physiological arousal if a lethal decision-making error is made. Finally, we expect increased HRMax during debriefing to be associated with overall reduced cognitive performance.

## Materials and methods

### Participants

Fifty-seven active-duty frontline police officers (7 female, 1 sex not reported, mean age: 32.8 ± 6.3; mean years of service: 7.2 ± 5.6) participated in a previously reported intervention study (Andersen et al., 2018). The original study conducted an *a priori* power analysis to determine a sufficient sample size for the original research goal, a within-subjects repeated-measure design, which indicated needing a total sample size of 24 participants and was exceeded at each time point of the intervention. The current observational study only assess data collected during the pre-intervention baseline evaluation session conducted in 2016. Participants were from a pool of approximately 750 frontline officers employed by a large urban police agency in Canada. Informed consent was provided by each participant before volunteering for the study. Participants were informed that they could withdraw at any point without penalty.

### Procedure

At the start of the session, officers were fitted with training versions of their usual police equipment including a baton, conducted electrical weapon (CEW), firearm, pepper spray, and full uniform. They were also fitted with a portable heart rate (HR) monitor that adhered to their skin via electrodes under their clothing (Bodyguard 2; FirstBeat Technologies LTD, Jyväskylä, FI). Officers completed four live-action reality-based scenarios that were designed and executed in a manner similar to police training and evaluation procedures. However, scenarios were delivered solely for research purposes to determine baseline rates of performance and physiological arousal. Scenarios were counterbalanced for each officer, with breaks in between scenarios.

#### Reality-based scenarios

The standard procedure of reality-based scenarios follows as such: the officer receives a briefing that mimics a call for service, during which they are given a radio dispatch containing limited information about the call. This can include suspect descriptions or details of the scene they will be attending (e.g., location, persons present). Officers then complete the scenario, during which they are required to: (1) form an understanding of the situation based on their perceptions, SA, and dispatch information, and (2) take action and make appropriate decisions, which can include physical tactics and positioning such as taking time and distance, giving verbal commands, and possibly employing a UOF option including lethal force if deemed necessary. After the scenario is complete, instructors typically conduct a post-incident debriefing where the officer can articulate their thought processes and provide rationale for their actions. In this way, the accuracy or “correctness” of decision-making can be judged based on the officers’ in-the-moment perceptions and SA, but which must nonetheless adhere to legal and organizational best practices (Di Nota et al., 2021a).

The scenarios used in this study were reflective of what officers at this particular agency are often exposed to during field duties. Scenario 1 involved helping a suicidal individual in crisis and Scenario 2 involved de-escalating a violent encounter during a reported break-and-enter (see Scenario Descriptions for more detail). For the current study, qualified police instructors provided participants with very brief description of the situation before each scenario began. Instructors blew a whistle to mark start and stop times of the scenario. Each scenario was followed by an instructor-led debriefing period that probed the officers’ SA and decision-making. However, for the purpose of the current study, instructors were given specific questions to evaluate officers’ memory and remain consistent across officers. The debrief began with the instructor asking the officer to provide an open-ended recall (i.e., “What did you see and hear?”), followed by specific probes for aspects of the scenarios the officer did not mention (i.e., “Then what did you do?,” “Did you see the backpack in the corner?,” “What was the suspect description provided during briefing?”). Importantly, instructors first gathered what the officer perceived and remembered in order to avoid engaging in leading questions. Officers’ participation in the current study was not part of their professional duties or training but instead served as baseline performance in a research study on police performance during stress (Andersen et al., 2018). Therefore, officers’ actions were not corrected following each scenario but rather were discussed and debriefed at the end of the research component (Andersen et al., 2024).

To avoid any researcher bias or confounds, the reality-based scenarios were designed by qualified and experienced UOF instructors with more than 10 years of experience designing occupationally relevant evaluation and training scenarios. These instructors were not part of the research team and were employed by the police agency from which the study sample was derived. In accordance with pedagogical principles for evidence-based police training (Di Nota et al., 2021a; Jenkins et al., 2020), each scenario was designed to be appropriately challenging in eliciting fundamental police skills of SA, physical tactics (e.g., positioning, maneuvering), and decision-making (i.e., selection of an appropriate force option, UOF implementation) and induce autonomic arousal that is comparable to encounters faced by officers in the field (Andersen et al., 2016; Arble et al., 2019; Baldwin et al., 2019). To further enhance ecological validity, scenarios were conducted at an empty school to allow for indoor and outdoor scenes ‘on location’. Props were used to enhance reality, including furniture and simulated weapons. Officers’ firearms were loaded with blank ammunition that mimics the sound of live fire, and actors present were police officers and trainers experienced in acting out critical incident scenarios.

For the current study, all procedures (briefing, scenario, debrief) were audio recorded, transcribed, and coded for cognitive performance. However, only two of the four completed scenarios met the criteria for addressing the current analyses and hypotheses: one excluded scenario did not require a lethal force decision, and cognitive performance scores could not be coded in the second because officers were not consistently asked to recall scenario details during debriefing. HR and demographic information were available for all 57 participants; however, audio recordings were missing for two participants in Scenario 1 and missing for 12 participants in Scenario 2.

#### Scenario descriptions

For Scenario 1, officers were called to a home to conduct a ‘wellness check’ (i.e., physically confirm an individual’s wellbeing) on a female as requested by her boyfriend. Entering the scenario, the officer did not know if the individual was experiencing a crisis, had a weapon, or would be violent. Thus, all police tools and options were available to the officer, and the decision about how to resolve the incident had to be based on the officer’s assessment of the situation. Upon approach to the doorway of the location, a male, who is the boyfriend of the person in crisis, can be heard yelling “Stop! Why are you doing this?” He was standing close to a female who was seated, slightly slumped over, with visible blood marks in a line across her upturned wrists. Upon arrival, the officer discovers the male holding a knife in his left hand as he takes it away from the female, whose wounds are self-inflicted. Upon verbal orders and further questioning by the officer, the male immediately dropped the knife, and the female state that she did not want to live any longer (i.e., indicating that she was suicidal). According to agency best practices and instructor criteria, the officer should prone all individuals on the ground, secure the weapon, and call for backup before approaching too closely. The scenario was designed such that the optimal lethal force decision was one of inhibition, meaning a ‘no-shoot’ decision was appropriate.

For Scenario 2, officers were called to a home for a reported break-and-enter. During briefing, a suspect was described to be seen kicking in a door wearing a specific-colored shirt; therefore, deciding to implement a UOF option may be necessary. Upon arrival, the officer discovers two men fighting—one in a housecoat holding a crowbar, who is the homeowner, and another in a shirt matching the suspect’s description. The homeowner’s wife is also present, hiding in the background, and can be heard yelling “Stop!” several times. Based on the briefing information, the officer must decipher who is the homeowner and who is the suspect. Given the involvement of a weapon, all police tools and options were available to the officer. Similar to Scenario 1, the homeowner is to immediately comply with officer commands, drop the crowbar, and all individuals should be laid on the ground away from the weapon and kept at a distance until backup is radioed in. The scenario was also designed as a ‘no-shoot’ decision, whereby deciding not to use lethal force was appropriate.

### Measures

#### Heart rate

Continuous physiological data were recorded at a rate of 1 Hz (1 recording/s) using Bodyguard 2 cardiovascular monitors (FirstBeat Technologies Ltd., Jyväskylä, FI). Monitors were worn by officers for the entire procedure, including during the briefings, scenarios, and debriefing periods. One adhesive electrode patch was applied to the officers’ skin below the left collarbone, and another was applied to the ribcage below the heart. Four cardiovascular measures were analyzed for the purpose of this study: (1) resting heart rate (HRRest), which was recorded at the beginning of the day and averaged over 5 min of seated rest; (2) maximum heart rate (HRMax) during the anticipatory briefing period before the scenario began; (3) HRMax during the scenario (i.e., between the start and stop whistles); and (4) debriefing HRMax experienced after the scenario. All HRMax values were 5-s averages centered on the maximum HR value achieved during each respective time period.

#### Cognitive performance

A coding scheme was created specific to each scenario to assess officers’ use of best practices in the scenario, officers’ post-incident memory of their initial perceptions and actions, and their understanding or sensemaking (i.e., correctly decipher who was the homeowner). It is important to highlight that “best practices” are defined as the policies and procedures that the police agency follows in accordance with Canadian legal statutes (i.e., they do not reflect the personal opinions of the researchers).

Accordingly, cognitive performance was operationalized by the successful execution of specific behaviors during the scenario (e.g., issuing verbal commands) and/or identification of specific items during the instructor-led debrief (e.g., recalling what the actors said) based on notes and transcriptions from audio-recordings. Items were coded as binary scores and grouped into a total of four composite variables, or when only one item existed, represented individually:

- *Perceptual memory*, assessing officers’ recall of visual and auditory scenario elements.
- *Procedural memory* of enacting previously trained best practices and complex sensorimotor skills during the scenario.
- *Action memory*, which is the officers’ memory of whether they implemented certain best practices.
- *Understanding* (represented individually), which assessed officers’ ability to build upon their perceptions of the environment and make sense of the dynamics of the situation.

Individual items included in composite scores are reported in Table 1 (see Supplementary materials for detailed descriptions). Items were coded through verification from transcriptions of the scenario (e.g., the officer gave verbal commands during the scenario, the instructor described the officer placed themselves too close to the actors and therefore used inappropriate positioning), as well as from notes from the instructor and research team (e.g., the instructor noted the officer drew pepper-spray as the force option). Officer memory was coded explicitly for their actions and later memory of what transpired in the scenario and not alternative choices they would have made in hindsight. For each scenario, individual items were summed and converted to a percentage score to reflect cognitive performance for each composite variable as well as for overall cognitive performance.

**Table 1:** Descriptive statistics and demographic summary.

Variable	*M*	SD	*n*	Range
Sex (Female, unknown)			49 male (7, 1)	
Age	32.80	6.29	56	23–47
Years of service	7.15	5.64	56	1.0–28.6
HRRest	76.73	10.48	57	55.44–100.79
Scenario 1				
Lethal decision-making errors			7	
Anticipation HRMax (bpm)	99.86	17.79	57	66.6–139.0
Scenario HRMax (bpm)	108.36	19.99	57	62.6–154.2
Debrief HRMax (bpm)	106.48	21.22	57	72.6–149.4
Perceptual memory	90.45	18.94	55	25–100
Heard actor speaking	96.40		54	
Reported seeing the knife	100		55	
Reported seeing the blood	87.30		55	
Reported seeing the wounds	76.40		55	
Procedural memory	68.33	20.37	55	25–100
Used correct UOF option	79.60		54	
Used radio follow up	67.30		55	
Gave verbal commands	100		55	
Used appropriate position	27.30		55	
Action memory	92.59	20.39	54	0–100
Memory of using radio follow up	88.90		45	
Memory of giving verbal commands	94.40		54	
Understanding the situation (binary)*	52.50	50.57	40	0–100
Cognitive performance score	79.55	12	55	50–100
Scenario 2				
Lethal decision-making errors			1	
Anticipation HRMax (bpm)	101.98	18.83	57	62.6–148.6
Scenario HRMax (bpm)	114.89	23.17	57	62.6–163.8
Debrief HRMax (bpm)	109.79	24.61	57	64.6–154.0
Perceptual memory	70.19	24.10	45	0–100
Suspect description	77.78		27	
Reported seeing the crowbar	97.67		43	
Reported seeing the backpack	31.71		41	
Saw/Heard female	77.78		45	
Procedural memory	54.26	28.5	45	0–100
Used correct UOF option	88.89		36	
Used radio follow up	62.22		45	
Interacted with female	33.33		45	
Used appropriate position	38.24		34	
Action memory (binary—memory of using radio follow up)	100	0	19	0–100
Understanding the situation (binary)*	57.78	49.9	45	0–100
Cognitive performance score	63.35	17.8	45	28.6–100

Cognitive performance scores reported in percent. M: Mean; SD: Standard deviation; n: sample size; HRRest: resting heart rate (reported in beats per minute); HRMax: maximum heart rate (reported in beats per minute); bpm: beats per minute. *Understanding the situation correctly is defined as the officer understanding that the male was attempting to help the suicidal woman, not hurt her (Scenario 1) and correctly identifying who was the homeowner (Scenario 2).

Individual items that were not explicitly addressed during the debrief (e.g., the officer was not asked about their memory of an aspect of the scenario) or which could not be ascertained from the transcript and notes (e.g., the use of force option selected was not explicitly stated or noted by the research team) were coded as missing values. Such instances are due to the applied research context, in that live-action reality-based scenarios used by law enforcement for training and evaluation purposes are subject to slight variations despite having pre-determined scripts and outcomes. For instance, the dynamic verbal interactions between actors and officers may slightly differ between individual trials or run-throughs based on what the officer says or does (e.g., an officer issues a verbal command before the actor has a chance to make their scripted statement). Although this is less controlled than typical lab-based experiments, live-action scenarios along with transcribed recordings provide a unique opportunity to examine episodic memory and cognition in policing in a real-world applied setting, with the detailed context of each scenario verifiable for (1) what truly occurred, even if diverging from the pre-determined script, and therefore (2) the accuracy of officer’s subsequent event memory. We have mitigated the impact of idiosyncratic differences in individual run-throughs by calculating overall cognitive performance for each run-through as a proportion of all available items and excluding the analysis of transcripts from a third scenario that featured inconsistent instructor debriefs and actor performance.

Consistent with previous reports on the current dataset (Andersen et al., 2018, Di Nota et al., 2021a; Chan et al., 2022), lethal force decision-making was coded as a binary outcome (correct/incorrect), with the correct decision for both scenarios being a ‘no-shoot’ response in alignment with the police agency’s best practices. As most participants did not make a lethal decision-making error (89 and 98%, respectively), there was an inflation of zero-coded actions. To assess the relationship between cognitive performance, physiological arousal, and making a lethal-force decision-making error (i.e., making a “shoot” response), making a lethal force error was coded separately (i.e., not included in composite cognitive performance measures). All items were coded by the lead and second authors and Cohen’s Kappa calculated to assess inter-rater reliability. This found good agreement for cognitive performance items for Scenario 1 (Cohen’s Kappa = 0.77, *p* < 0.001) and Scenario 2 (Cohen’s Kappa = 0.81, *p* < 0.001).

### Statistical analyses and design

Statistical analyses were conducted using SPSS (Version 20; IBM Corp, 2022) (Version 4.4.1; R Core Team, 2024) and R Studio (Version 2024.4.2.764, Posit Team, 2024). First, physiological arousal before, during, and after each scenario was compared to officers’ own baseline HRRest to examine if the scenarios elicited a physiological response. Paired samples t-tests revealed significant differences in HRMax between scenarios (*p*s ≤ 0.008), therefore two repeated measures ANOVAs were run evaluating differences in HRRest and HRMax before, during, and after each scenario (4 levels) with age, sex, and years of experience included as covariates. Effect sizes are reported using partial eta squared (Cohen, 2013) and *post hoc* tests adjusted with Bonferroni corrections.

Correlation matrices were generated for each scenario to explore the relationships between physiology, lethal force errors, and individual cognitive performance items. Due to cognitive variables being non-normally distributed (see Supplementary materials), Spearman correlations were chosen. As an exploratory analysis, we also wanted to examine the relationship between stress physiology, lethal force errors, and composite scores of cognitive performance using a series of linear mixed effect models. Linear mixed-effects models are robust to data variability and can examine relationships across both scenarios simultaneously, which is important due to the limited instances of lethal force errors and non-normality seen in this data. To examine the relationship between stress physiology and cognitive performance, five models were run predicting overall cognitive performance as well as composite perceptual memory, procedural memory, action memory, and understanding scores by anticipatory HRMax, HRMax during the scenario, and HRMax during debriefing. Age, sex, years of experience, HRRest, and making a lethal decision-making error were entered as covariates, and all models included a random intercept for participant. To examine the relationship between lethal force decision-making, cognitive performance, and stress physiology, a linear mixed-effect model was run with overall cognitive performance, anticipatory HRMax, HRMax during the scenario, and HRMax during debriefing included as predictors, HRRest as a covariate, and participant as a random participant. Given the limited number of errors (*n* = 8), a simpler full model had to be examined without including age, sex, and years of experience. Given that understanding and lethal force errors were single binary scores, these models were run as mixed-effect logistic regressions conducted using the logit function. All models were conducted in a backwards stepwise fashion, removing non-significant predictors to find the most parsimonious model. Significance criteria was set at *p* < 0.05 and model comparisons completed using AIC. Models were conducted using the lme4 package (version 1.1.35.5; Bates et al., 2015) with *p*-values approximated using lmerTest (version 3.1.3; Kuznetsova et al., 2017).

## Results

A descriptive summary of the study findings can be found in Table 1, including average HRRest, HRMax, and all cognitive performance scores.

### Scenario length and use of lethal force

Seven of 57 participants made a lethal force decision-making error (i.e., shooting the male actor) during Scenario 1. Mean duration for the briefing was 39.9 s ± 12.6. The scenario lasted an average of 39.7 s ± 21.7, and the debrief was on average 179.0 s ± 70.5. One of 57 participants made a lethal force decision-making error during Scenario 2. Mean duration for the briefing was 48.6 s ± 13.2. The scenario lasted an average of 50.6 s ± 27.9, and the debrief was on average 141.8 s ± 53.3. Police officers did not make an error in more than a single scenario; therefore, we did not find any order effects in how making a lethal decision-making error impacted later performance.

### Physiological arousal and scenario performance

**Figure 1:**
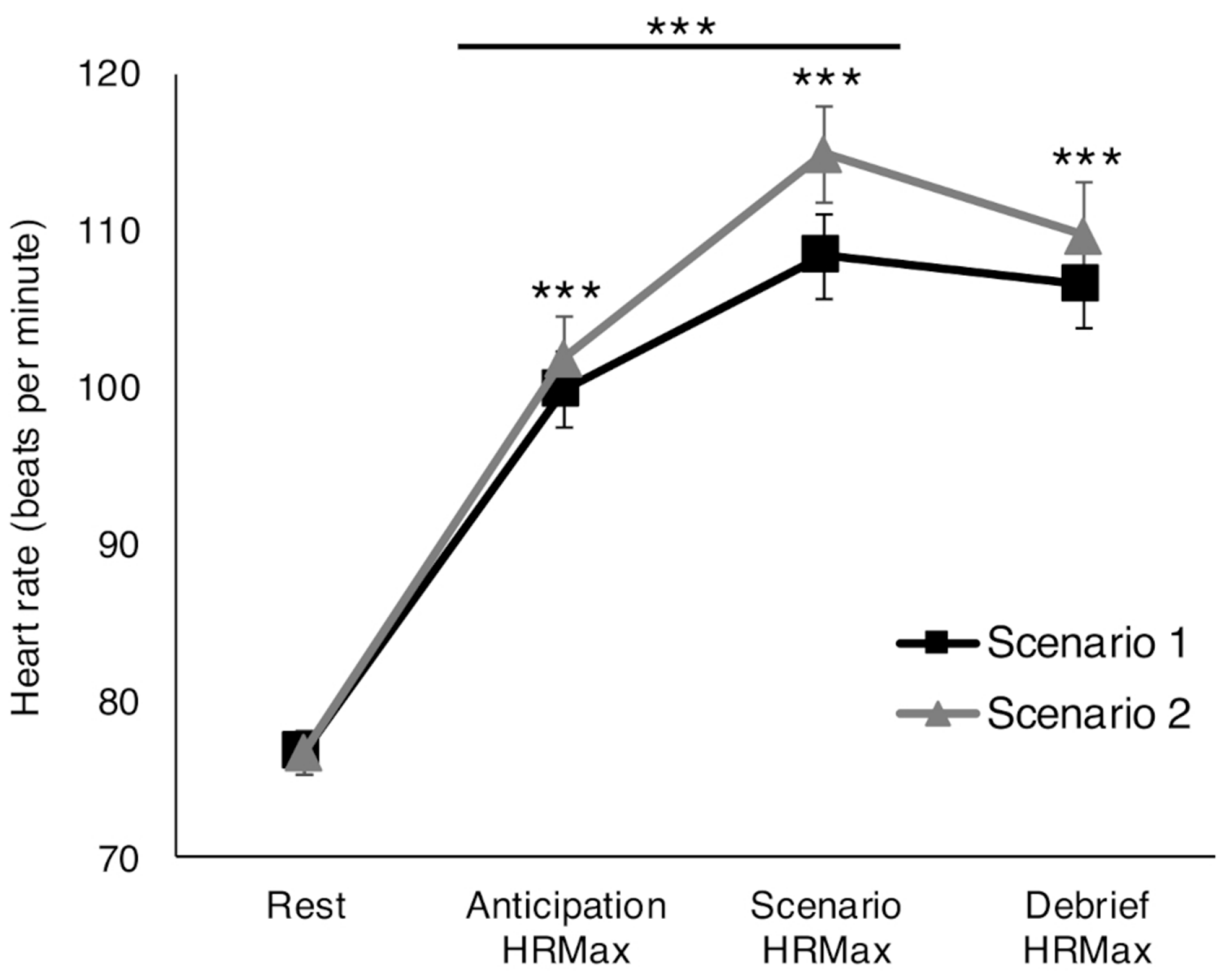
Physiological arousal in response to reality-based scenarios. Both scenarios significantly elevated maximum heart rate (HRMax) compared to rest at all time points: before the start of the scenario, during the scenario, and during the post-scenario debrief periods. HRMax was significantly higher during the scenarios compared to the anticipatory periods. For Scenario 2, HRMax was also significantly higher during the debrief period relative to anticipation. ***p_Bonf_ ≤ 0.001.

For both scenarios, HR was significantly elevated at all time points relative to rest (Scenario 1: *F*(3, 156) = 2.791, *p* = 0.042, *η_p_*^2^ = 0.051; Scenario 2: *F*(3, 156) = 3.107, *p* = 0.028, *η_p_*^2^ = 0.056). Further, HRMax was higher during both scenarios relative to anticipation periods (*p_Bonf_* ≤ 0.001) and higher during Scenario 2 debrief relative to anticipation (*p_Bonf_* = 0.030; Figure 1).

During both scenarios, officers were able to perceive and subsequently recall highly salient cues attributed to the central target individuals, including verbal interactions (96.4%), weapons (knife: 100%, crowbar: 97.7%) and blood (87.3%). However, perceptual memory was lower for more detailed (wounds: 76.4%), peripheral visual (backpack: 31.7%), and auditory cues (vocals from a woman hiding: 77.8%). Despite reporting hearing the actors during the scenarios, officers did not always interact with them, as only 33.3% of officers engaged with the female in Scenario 2. Furthermore, many were unable to articulate a correct understanding of each situation: only 52.5% of officers correctly understood that the male was assisting the suicidal female in Scenario 1 and 57.8% of officers correctly identified the homeowner in Scenario 2.

Officers’ procedural memory during both scenarios was moderate or high for issuing verbal commands (100%), radio communications (Scenario 1: 67.3%; Scenario 2: 62.2%), and identifying the appropriate force option (Scenario 1: 79.6%; Scenario 2: 88.9%). However, instructor feedback revealed poor positioning (Scenario 1: 27.3%; Scenario 2: 38.2%). Officers were also able to recall their own actions with a high degree of accuracy (Scenario 1: 92.6%; Scenario 2: 100%).

### Correlation analyses

Significant results are reported below (see Supplementary materials for full correlation matrices).

#### Scenario 1

For Scenario 1, age was negatively related to HRMax during the scenario (*ρ* = −0.314, *p* = 0.018, *n* = 56) and years of service was negatively correlated with HRMax during the debrief (*ρ* = −0.273, *p* = 0.042, *n* = 56), such that older and more experienced officers exhibited lower arousal. Years of service was also negatively correlated with procedural memory for UOF option (ρ = −0.367, *p* = 0.007, *n* = 53) and positively correlated with action memory for their own verbal commands (*ρ* = 0.343, *p* = 0.011, *n* = 54). This suggests that more experienced officers had poorer use of best practices regarding the UOF option they employed, but better recollection of their verbal commands during the scenario.

Higher anticipatory HRMax before the start of the scenario was positively related to understanding (*ρ* = 0.453, *p* = 0.003, *n* = 40). Higher HRMax during the scenario was negatively related to lethal decision-making errors (*ρ* = −0.367, *p* = 0.005, *n* = 57). Higher HRMax during the debrief period was negatively associated with procedural memory for using the radio during the scenario (ρ = −0.280, *p* = 0.039, *n* = 55) as well as post-scenario action memory for radio use (ρ = −0.310, *p* = 0.038, *n* = 45) and issuing of verbal commands (ρ = −0.288, *p* = 0.035, *n* = 54). Overall, this suggests that experiencing higher physiological arousal before and during the scenario increases situational awareness and is related to fewer errors in lethal decision-making. Additionally, higher arousal post-scenario is related to reduced memory for one’s verbal actions.

Several negative correlations were revealed for lethal force errors (12.3%). Officers that made lethal force errors scored lower in perceiving actor verbals (*ρ* = −0.356, *p* = 0.008, *n* = 54), perceiving the female actor’s wounds (ρ = −0.430, *p* = 0.001, *n* = 55), and action memory for their own verbal commands (*ρ* = −0.628, *p* < 0.001, *n* = 54). Thus, lethal decision-making errors committed during critical incident scenarios significantly degrade subsequent memory recall for perceptual and action-related details.

#### Scenario 2

Age and years of service were negatively related to HRMax before (experience: *ρ* = −0.269, *p* = 0.045, *n* = 56), during (age: ρ = −0.310, *p* = 0.002, *n* = 56; experience: ρ = −0.304, *p* = 0.023, *n* = 56), and after the scenario (age: ρ = −0.275, *p* = 0.041, *n* = 56). Similar to Scenario 1, this suggests lower arousal in older and more experienced officers.

Higher HRMax before (ρ = 0.374, *p* = 0.024, *n* = 36) and during the scenario (ρ = 0.477, *p* = 0.003, *n* = 36) was positively associated with procedural memory for using the correct UOF option. However, anticipatory HRMax was negatively associated with procedural memory for positioning (ρ = −0.512, *p* = 0.002, *n* = 34). Together, these findings suggest that experiencing higher physiological arousal before or during the scenario can promote better memory for using UOF best practices but hinder spatial processing that is central to police tactics and operations.

Procedural memory for using the correct force option was positively related to perceiving the target weapon (ρ = 0.560, *p* < 0.001, *n* = 34) and peripheral presence of a female bystander (ρ = 0.373, *p* = 0.025, *n* = 36). However, using the correct force option was negatively associated with lethal force decision-making errors (ρ = −0.478, *p* = 0.003, *n* = 36) of which there was only 1 out of 57 cases. Therefore, officers who more accurately perceived the target weapon and actors in play were more likely to use an appropriate force option and less likely to make a lethal UOF error. Similarly, whether officers perceived the female bystander was positively correlated to overall understanding of the situation (ρ = 0.301, *p* = 0.045, *n* = 45) and procedural memory for engaging with the female (ρ = 0.378, *p* = 0.010, *n* = 45) but negatively related to procedural memory for using the radio (ρ = −0.306, *p* = 0.041, *n* = 45). Thus, perceiving relevant target and peripheral cues was positively associated with forming more complete understanding. However, due to the time-constraints of these brief simulations, communicating with the female bystander may have taken priority over radio communications.

### Linear mixed-effect models

Of the planned mixed-effect models, all were found to have significant predictors except for procedural memory (see Table 2 for a summary and Supplementary materials for full models). The best model for overall cognition had lethal force error, sex, and anticipatory HRMax as predictors (AIC = 817.20) which was a better fit than the full model (AIC = 807.83), however only sex and making a lethal force error were significant. This suggests that officers who did not make a lethal force error and those that were female had better cognitive performance more broadly in the scenarios.

**Table 2:** Linear Mixed-Effect Models Summary: Associations Between Predictor and Dependent Variables.

Dependent variable	Predictor	*B*	*SE*	*CI*	*t or z*	*p*
Overall cognitive performance	Anticipation HRMax	−3.16	1.78	[−6.60, 0.29]	−1.78	0.079
	Female	10.24	4.72	[1.09, 19.38]	2.17	0.033
	Lethal force error	−14.70	6.22	[−26.76, −2.64]	−2.36	0.020
Perceptual memory	Age	−7.27	3.76	[−14.52, −0.02]	−1.93	0.056
	Female	13.96	6.71	[1.01, 26.90]	2.08	0.040
	Years of experience	5.39	3.84	[2.02, 12.79]	1.40	0.164
	Lethal force error	−17.63	8.55	[−34.11, −1.14]	−2.06	0.042
Action memory	Anticipation HRMax	2.18	3.89	[−5.10, 9.46]	0.56	0.577
	HRMax	−4.41	3.79	[−11.51, 2.70]	−1.16	0.250
	Debrief HRMax	−5.03	2.64	[−9.97, −0.85]	−1.91	0.061
	Age	−3.94	3.13	[−9.80, 1.93]	−1.26	0.213
	Female	9.44	5.38	[−0.63, 19.52]	1.76	0.084
	Years of experience	1.93	3.20	[−4.05, 7.92]	0.60	0.548
	Lethal force error	−29.32	7.13	[−42.67, −15.97]	−4.11	>0.001
Understanding	Anticipation HRMax	0.51	0.26	[0.17, 1.05]	1.96	0.049
	Female	1.82	0.82	[0.39, 3.76]	2.22	0.026
Lethal force error	HRMax	−2.86	0.87	[−8.01, −1.46]	−3.29	0.001
	Debrief HRMax	1.91	0.72	[0.71, 6.00]	2.67	0.008
	Cognitive performance	−0.06	0.03	[−0.14, 0.04]	−1.77	0.076

Each dependent variable represents its own linear regression analysis. See Supplementary materials for reports of full models. HRMax: Maximum heart rate. *p < 0.05; **p < 0.01; ***p < 0.001.

The best model for perceptual memory had lethal force error, sex, age, and years of experience as predictors (AIC = 878.08) which was a better fit than the full model (AIC = 868.05). Being female and not making a lethal force error was associated with improved perceptual memory. The best model for action memory had lethal force error, sex, age, years of experience, anticipatory HRMax, HRMax in the scenario, and HRMax during debriefing as predictors (AIC = 579.63) which was a better fit than the full model (AIC = 577.84). However, only making a lethal force was a significant predictor, suggesting those who made a lethal force error during the scenario had reduced memory for their other actions.

The best model for understanding the scenario had anticipatory HRMax and sex as significant predictors (AIC = 112.40) which was a better fit than the full model (AIC = 117.47), suggesting officers who were female or had lower HRMax before the scenario had better overall understanding. Finally, the best model for making a lethal force error had HRMax during the scenario and debriefing as significant predictors (AIC = 37.98) which was a better fit than the full model (AIC = 40.64). This suggests that officers who had lower HR during the scenario were more likely to make a lethal force error, which was associated with elevated HR during debriefing.

**Figure 2:**
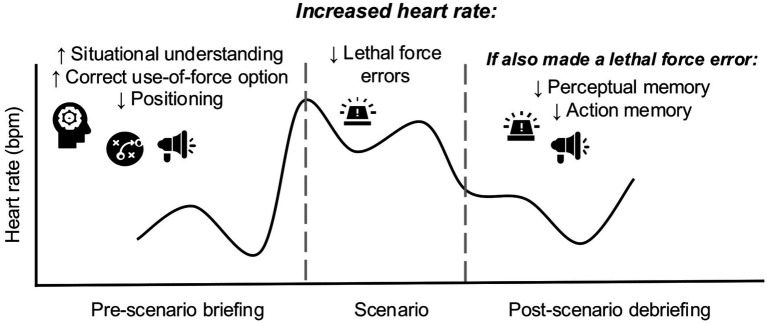
Visual summary of significant results. Increased heart rate during the pre-scenario briefing was associated with increased situational understanding during the scenario, as well as increased procedural memory for selecting the correct use of force option. However, it was also related to decreased procedural memory for using the best positioning. Increased heart rate during the scenario was associated with making fewer lethal force errors. Making a lethal force error was associated with a higher heart rate during the post-scenario debriefing and reduced memory for perceptual details and officer’s own actions in the scenario.

Overall, these results align with our correlation analyses, suggesting an adaptive role of anticipatory physiological arousal on situational awareness. They are also consistent with the findings that making a lethal force error is associated with lower HR during the scenario, as well as higher HR afterwards and later degradation of memory. See Figure 2 for a summary of significant results.

## Discussion

The current study provides evidence for a nuanced relationship between stress physiology and officers’ cognitive performance when completing reality-based scenarios involving persons experiencing a mental health crisis. Greater increases in HR before and during scenarios were associated with increased situational understanding, appropriate use of lethal force options, and fewer lethal decision-making errors. However, this was at the expense of officers using the best positioning and therefore not providing enough time and distance. Lower arousal during the scenario was associated with making lethal force decision-making errors, which led to subsequent impairments in post-incident memory for perceptual information and the officer’s own actions. Older and more experienced officers showed lower arousal, and female officers showed greater cognitive performance. These findings are discussed in the context of current basic and applied literature, as well as their implications for training procedures in critical incidents and crisis intervention.

### Anticipatory physiological arousal both disrupts and enhances cognitive performance

Increased physiological arousal before and during critical incident scenarios was associated with improvements in some aspects of cognition but impairments in others. These findings are consistent with both basic and applied police research, showing differential effects of physiological stress responses on cognitive performance (Shields et al., 2017; Arble et al., 2019). Moderate arousal appeared to play an adaptive role by promoting SA and improving lethal force decision-making accuracy. This may indicate increased vigilance and is consistent with previous findings that a constrained elevation in HR is associated with optimal lethal force decision-making (Arnetz et al., 2009). However, if HR is too elevated, perceptual distortions and cognitive and lethal force errors may occur (Baldwin et al., 2022). Thus, reported decrements to positioning may be accounted for by stress-induced perceptual distortions, such as tunnel vision. Here, attention is directed towards critical external details (e.g., weapon presence) at the expense of self-centered or spatial information (Novy, 2012). Spatial processing has previously been shown to be sensitive to stress in healthy and clinical populations, as well as in officers when completing live-action scenarios (Ellena et al., 2022; Marlatte et al., 2022; Lewinski et al., 2014). This may be an area for targeted improvement, especially during high-stress encounters.

### Older and more experienced officers demonstrate reduced engagement

As expected, we found significantly elevated HR before, during, and after critical incident scenarios relative to rest, indicative of physiological arousal and engagement with the scenarios. However, we observed that older and more experienced officers had lower HR during the scenario and debrief periods, consistent with previous research (Baldwin et al., 2022, but see Vickers and Lewinski, 2012; Renden et al., 2015; Mangels et al., 2020). Being more experienced was also related to being less likely to use the correct force option suggesting that older, more experienced officers may have been less engaged during these non-mandatory professional exercises (i.e., a research study). Increased training and on-the-job experience, therefore, do not compensate for a lack of task engagement and resultant arousal, which is necessary for optimal cognitive performance.

### Female officers demonstrate better cognitive performance

To our knowledge, this is the first study to report sex differences in police cognition during critical encounters. We found that female officers showed better overall cognitive performance, as well as improved perceptual memory and situational understanding, compared to male officers. This is consistent with basic science findings in episodic memory, where women show more detailed and accurate memory and focus more on the overarching meaning (Grysman and Hudson, 2013). Previous applied research showed that female officers self-reported higher stress before navigating critical incidents, which was independent of scenario performance (Di Nota et al., 2024). This higher anticipatory stress may actually be enhancing cognition when completing scenarios, leading to greater situational engagement and improved memory. Future studies should examine the interaction of stress and gender to better understand its potentially beneficial relationship to different aspects of scenario performance.

### Lethal decision-making errors as a predictor of post-incident memory

Committing a lethal decision-making error was the strongest predictor of poor cognitive performance. However, cognitive performance itself did not significantly predict the likelihood of making a lethal force error. This indicates that while officers who made a lethal force decision-making error generally exhibited lower cognitive performance, many others with similarly poor performance did not make such errors. Consequently, reduced cognitive performance alone cannot fully explain the occurrence of lethal force errors. Instead, lower arousal during the scenario emerged as a significant predictor of making a lethal force error, suggesting it may play a critical role as well. Physiological arousal is therefore an important target for intervention in reducing lethal force errors, particularly for achieving moderate levels of arousal that are adaptive (Andersen et al., 2016, 2018, 2024).

The Unease Modulation Model (UMM), a theory of stress relevant to law enforcement personnel, offers a framework to understand how decision errors can impact cognitive functioning afterwards (Arpaia and Andersen, 2019). Specifically, a mistake involving the use of lethal force can divert an officer’s focus from the immediate task to their internal feeling of unease. Because the officer is no longer focused on the skills they need to resolve the event, but rather on their physiological stress response and cognitions about potential occupational repercussions of making a mistake, performance can become impaired. Our findings are aligned with the UMM in that making a lethal force error was associated with increased HR after the scenario, an indicator of unease. As stress during recall can lead to memory impairments, the accumulation of error-induced stress post-scenario could account for the observed impediments in recollection (Shields et al., 2017).

The fact that perceptual and action memory were selectively impaired by lethal decision-making errors has significant implications for real-world encounters that require long-term recollection for legal purposes (e.g., testimony or investigation of officer-involved shootings). This is of particular importance in situations involving individuals with mental illness, where rates of UOF are higher and reliance on police testimonies may be greater (Fyfe, 2000). The current findings highlight the importance of reducing stress physiology during post-incident debrief procedures to enhance memory preservation.

### Training considerations

Officers generally demonstrated good verbal communication skills. and those who perceived relevant threat-related cues were more likely to implement best practices and select the appropriate UOF option. However, officer positioning was overall quite poor. Experts have identified creating time and distance when interacting with persons in crisis as de-escalation and non-escalation safety strategies, as well as central aspects of police-specific SA (Huey et al., 2021; Huhta et al., 2023; Di Nota et al., 2021c). Because of this, experts advocate for the incorporation of time and distance training for police officers who engage with individuals in crisis. A model for this specific type of training (i.e., *Decision Model for Police Encounters*) is available in the literature, along with policy recommendations to enhance evidence-based policing (Huey et al., 2021). Given the lack of standardization in training and performance measurement across the policing profession broadly, it is unclear whether and how time and distance are trained both conceptually and practically (Di Nota and Huhta, 2019). The findings from this study reveal the prevalent use of inadequate positioning, highlighting the pressing need for reform and standardization of current police training models.

Similarly, increased anticipatory physiological arousal may hinder officers’ *self*-awareness in space, despite improving other aspects of cognitive performance. Recent studies have identified self-awareness, defined as the awareness of one’s impact on situational outcomes, as one of several elements of overall SA (Huhta et al., 2022, 2023). Notably, highly experienced officers and police instructors report self-awareness more frequently compared to novice trainees (Huhta et al., 2023). Further, there are empirically based police interventions that train adaptive self-regulation skills – including breathing and body position – for the purpose of modulating physiological stress responses that might otherwise inhibit good decision-making during encounters with persons in crisis (for example, see Andersen et al., 2018, 2024). Employing self-regulation skills before and during scenarios have been shown to promote SA, verbal communication and de-escalation, physical tactics and positioning, as well as reduce lethal force decision-making errors (Andersen et al., 2016, 2018). The current findings suggest that adaptive self-regulation skill training can be extended to examine their potential benefits to developing officers’ self-awareness more broadly, as well as target post-scenario physiological arousal to possibly offset the negative impact of error-induced stress on officers’ memory.

Current models of crisis intervention training (CIT) aim to reduce the probability of UOF during incidents involving individuals with mental illness, using scenario-based training that promotes effective communication and de-escalation (Pelfrey and Young, 2020). Our results suggest that integrating adaptive physiological self-regulation skills into current models may improve officers’ overall SA. This may lead to more accurate situational understanding and decision-making, which could in turn reduce the use of lethal force in mental health crises. CIT has previously been criticized for being a systemic rather than an individual intervention (Watson et al., 2008b). It therefore may be an important future direction to incorporate physiological stress modulation tailored to the individual officer to improve the outcomes of police interactions with persons experiencing a mental health crisis (Andersen et al., 2024).

### Moderate arousal levels is associated with optimal cognitive performance

It has long been theorized outside of police contexts that the impact of stress on cognitive performance depends on achieving an optimal level of arousal (Yerkes and Dodson, 1908). This relationship between arousal and cognition follows an inverted U-shape, wherein moderate levels of arousal improves performance but further increases can lead to impairments. The lower heart rates observed in older officers and those who made a lethal decision-making error may reflect earlier stages of this curve, wherein moderate arousal levels necessary for optimal cognitive performance were not achieved. In contrast, younger and female officers may have been closer to optimal arousal levels, leading to better performance. This inverted U-shaped relationship arises from several brain regions being sensitive to the transient neurotoxic effects of stress (e.g., the hippocampus, implicated in memory and spatial processing, and the prefrontal cortex, which is critical for top-down control of flexible behavior in-the-moment), with each region differing in their degree of sensitivity (Arnsten, 2009; Arnsten, 2015). As a result, the same stressor may lead to moderate activation in one region, improving performance in related cognitive processes, and overactivation in another, leading to impairments in other aspects of cognition. This may explain why the same level of arousal seen in officers before the scenarios was adaptive for higher-level cognitive processing, leading to improved understanding and choice of use-of-force option, but exceeded optimal levels for spatial processing, resulting in impairments.

### Limitations

Inherent in all applied research, our study has limitations, including sample size constraints and the trade-off between realism and experimental control. However, as the scientific literature highlights, all studies, including experimental ones, face constraints on generality (COG; Simons et al., 2017). The COGs inherent in applied, contextualized research are balanced by the recognition that such research is essential for establishing legitimacy in psychological science (Andersen, 2024). This is especially true in understanding police interactions with individuals experiencing a mental health crisis. With this in mind, the COGs in this study indicate that our results may be most relevant to the experiences of Canadian officers in medium-to-large urban agencies, such as those in our sample. However, we also note that while there are some differences in police best practices across agencies, the policies and best practices regarding lethal force decision-making remain largely consistent with those of international police organizations. Thus, we recommend the reader to keep these limitations and benefits in mind while interpreting the findings of this study.

Further considerations include the following: data were coded from notes and transcriptions from audio-recorded scenarios, which are less detailed than other methods (e.g., video recordings) but were used following agency allowances. Additionally, not all officers experienced the exact same stimuli due to slight differences in scenario execution by the actors or in debriefing procedures, which was mitigated in multiple ways (see *Cognitive Performance* in Methods). The noted heterogeneity is common in applied research settings, as stringent experimental control of all variables is often not possible and would undermine the ecological validity of police training in field contexts as it does not reflect the dynamic interactions police are required to navigate in the real world.

Similarly, different scenarios elicit different responses in individual officers; therefore, it is not possible ensure a wide range of lethal decision-making errors. We believe it is not ethical to experimentally manipulate officers into making lethal force errors to inflate the number of errors to increase statistical reliability (Andersen, 2024). While this may lead to higher degrees of variability in model estimates, as seen here, we mitigated the impact that rare outcomes have on statistical analyses by using approaches for zero-inflated variables and analyzing across multiple scenarios simultaneously. Given the limited sample size and number of scenarios, these results offer exploratory insights into the nuanced relationship between the physiological stress and police memory and cognition, which merits further study. Although correlational in nature, these analyses do provide some understanding to the temporal contingency of how stress impacts aspects of cognition in realistic, applied settings and lays the foundation for future work in this area.

## Conclusion

We found a complex relationship between stress physiology and officers’ cognitive performance during reality-based scenarios involving individuals in mental health crisis. An optimal amount of arousal before and during scenarios was found to be necessary for the best cognitive performance but comes at the cost of officers’ spatial awareness. Reduced arousal during scenarios can lead to lethal force errors, which are associated with increased arousal after the scenario and can result in impaired memory. Our findings reinforce existing empirical research showing that age and years of experience do not automatically result in better skills and performance, and reveal that female officers display better cognitive performance and situational understanding, a novel finding which future studies should explore further.

These findings highlight the need for training that integrates physiological self-regulation and targets officer’s self-awareness. We also urge policy makers and police agencies to revise existing training models to include time and distance considerations as methods of de-escalation and non-escalation. Together, these changes can improve officers’ cognitive performance, reduce lethal force errors, and mitigate stress-related impairments for all involved, ultimately improving outcomes during encounters with individuals in a mental health crisis.

## Data Availability

The datasets presented in this article are not readily available because of research ethics agreements protecting the privacy and confidentiality of participants who were active-duty police officers at the time of data collection. Aggregate or de-identified data can be made available upon request from the corresponding author with ethics board approval. Requests to access the datasets should be directed to Judith Andersen, judith.andersen@utoronto.ca.
