# Supplementary material for: Physiological stress differentially impacts cognitive performance during—and memory following—simulated police encounters with persons experiencing a mental health crisis
Source: Front Psychol. 2025 Mar 18;16:1549752. doi: 10.3389/fpsyg.2025.1549752 (PMC11959019; doi:10.3389/fpsyg.2025.1549752)
Supplement: Supplementary file 2 [file Supplementary_file_1.docx]

**Descriptions of Individual Cognitive Performance Items**

For Scenario 1, eleven items were included within the following composite variables.

Perceptual memory items assess the officer’s accurate recollection of key perceptual details of the scenario: (1) accurately recalling hearing the actors speak; (2) accurately recalling seeing the knife held by the male actor; (3) accurately recall seeing blood on the female actor; and (4) accurately recalling seeing wounds on the female actor. Procedural memory items assess the officer’s employment of best practices during the scenario which have been trained through prior occupational experience: (5) employing the correct level of force when interacting with someone with a knife (i.e., first drawing a firearm); (6) using appropriate positioning (i.e., using time and distance and therefore not rushing in towards the actors), (7) giving clear verbal commands to the actors, such as to prone on the ground or drop the weapon; and (8) using their radio to communicate with dispatch about the situation and necessary next steps. Action memory items included the officer’s accurate recollection of if they employed certain best practices: (9) accurate recall of if they did use their radio to communicate with dispatch, and; (10) accurate recall of if they did give verbal commands to the actors. Understanding the situation was a binary item assessing the officer’s accurate interpretation of the scenario, such that (11) they correctly identified that the man holding the knife was helping the woman, who was suicidal and had inflected the wounds herself.

For Scenario 2, ten items were included within the following composite variables. Perceptual memory items assess the officer’s accurate recollection of key perceptual details of the scenario: (1) accurately recalling the suspect description given during initial briefing (i.e., that the suspect was wearing a specific-coloured shirt); (2) accurately recalling seeing the crowbar held by the homeowner; (3) accurately recalling seeing a backpack sitting by the door, and; (4) accurately noticing a female actor who was hiding in the background of the scenario. Procedural memory items assess the officer’s employment of best practices during the scenario which have been trained through prior occupational experience: (5) employing the correct level of force when interacting with someone with a crowbar (i.e., first drawing a firearm); (6) using appropriate positioning (i.e., using time and distance and therefore not rushing in towards the actors); (7) giving clear verbal commands to the female actor, such as asking for her to come out of hiding or asking if she is okay, and; (8) using their radio to communicate with dispatch about the situation and necessary next steps. Action memory assesses the officer’s accurate recollection of if they employed certain best practices, to which there was a single item identifying if the officer (9) accurately recalled using their radio to communicate with dispatch. Understanding the situation was a binary item assessing the officer’s accurate interpretation of the scenario, such that (10) they correctly identified that the homeowner was the man in the housecoat holding the crowbar and the intruder was the man wearing the shirt that matches the suspect description.

**Tests of Normality**

Most of the cognitive composite variables are not normally distributed, therefore non-parametric correlations were used.

|  | **Kolmogorov-Smirnov^+^** | | | **Shapiro-Wilk** | | |
| --- | --- | --- | --- | --- | --- | --- |
|  | **Statistic** | **df** | **Sig.** | **Statistic** | **df** | **Sig.** |
| Age | .158 | 56 | .001 | .928 | 56 | .002 |
| Years Experience | .152 | 56 | .002 | .828 | 56 | <.001 |
| HRRest | .059 | 57 | .200^*^ | .988 | 57 | .842 |
| S1AntMax | .068 | 57 | .200^*^ | .980 | 57 | .457 |
| S1Max | .050 | 57 | .200^*^ | .993 | 57 | .987 |
| S1DebriefMax | .099 | 57 | .200^*^ | .954 | 57 | .031 |
| S2AntMax | .072 | 57 | .200^*^ | .987 | 57 | .793 |
| S2Max | .070 | 57 | .200^*^ | .980 | 57 | .453 |
| S2DebriefMax | .105 | 57 | .181 | .961 | 57 | .067 |
| S1_Perception | .457 | 55 | <.001 | .562 | 55 | <.001 |
| S2_Perception | .220 | 45 | <.001 | .882 | 45 | <.001 |
| S1_Understanding | .351 | 40 | <.001 | .636 | 40 | <.001 |
| S2_Understanding | .379 | 45 | <.001 | .628 | 45 | <.001 |
| S1_ProcMem | .265 | 55 | <.001 | .867 | 55 | <.001 |
| S2_ProcMem | .157 | 45 | .007 | .930 | 45 | .010 |
| S1_ActionMem | .512 | 54 | <.001 | .407 | 54 | <.001 |
| S2_ActionMem | . | 19 | . | . | 19 | . |
| S1_CogPerf | .151 | 55 | .003 | .947 | 55 | .017 |
| S2_CogPerf | .084 | 45 | .200^*^ | .978 | 45 | .553 |

| + Lilliefors Significance Correction  * This is a lower bound of the true significance. |
| --- |
|  |

**Linear Mixed-Effect Models: Full Models**

| **Dependent Variable** | **Predictor** | ***B*** | ***SE*** | ***CI*** | ***t or z*** | ***p*** |
| --- | --- | --- | --- | --- | --- | --- |
| *Overall Cognitive Performance* | Anticipation HRMax | -2.77 | 3.68 | [-9.72, 4.18] | -0.75 | .454 |
|  | HRMax | -1.91 | 3.66 | [-8.82, 5.00] | -0.52 | .604 |
|  | Debrief HRMax | 0.28 | 2.69 | [-4.79, 5.35] | 0.10 | .917 |
|  | Age | -0.96 | 2.87 | [-6.37, 4.44] | -0.34 | 0.738 |
|  | Female | 10.01 | 4.99 | [0.59, 19.42] | 2.00 | .048 |
|  | Years of Experience | -0.12 | 2.94 | [-5.66, 5.42] | -0.04 | .967 |
|  | Lethal Force Error | -17.63 | 7.27 | [-31.33, -3.92] | -2.43 | .017 |
|  | HR Rest | 1.48 | 2.30 | [-2.86, 5.81] | 0.64 | .522 |
| *Perceptual Memory* | Anticipation HRMax | -1.17 | 5.16 | [-10.91, 8.56] | -0.23 | .821 |
|  | HRMax | -1.45 | 5.14 | [-11.14, 8.24] | -0.28 | .778 |
|  | Debrief HRMax | 0.07 | 3.77 | [-7.04, 7.18] | 0.02 | .986 |
|  | Age | -7.42 | 4.02 | [-15.00, 0.17] | -1.84 | .069 |
|  | Female | 13.96 | 7.00 | [0.75, 27.17] | 1.99 | .049 |
|  | Years of Experience | 4.70 | 4.12 | [-3.07, 12.47] | 1.14 | .257 |
|  | Lethal Force Error | -20.66 | 10.19 | [-39.89, -1.44] | -2.03 | .046 |
|  | HR Rest | 0.52 | 3.22 | [-5.56, 6.59] | 0.16 | .873 |
| *Procedural Memory* | Anticipation HRMax | -10.48 | 5.60 | [-21.04, 0.08] | -1.87 | .064 |
|  | HRMax | 3.18 | 5.57 | [-7.33, 13.68] | 0.57 | .570 |
|  | Debrief HRMax | 2.49 | 4.09 | [5.22, 10.20] | 0.61 | .544 |
|  | Age | 6.73 | 4.36 | [-1.49, 14.95] | 1.54 | .126 |
|  | Female | 2.80 | 7.59 | [-11.52, 17.12] | 0.37 | .713 |
|  | Years of Experience | -4.24 | 4.47 | [-12.66, 4.19] | -0.95 | .345 |
|  | Lethal Force Error | -7.34 | 11.05 | [-28.18, 13.49] | -0.67 | .508 |
|  | HR Rest | 2.55 | 3.49 | [-4.04, 9.13] | 0.73 | .468 |
| *Action Memory* | Anticipation HRMax | 2.23 | 3.98 | [-5.18, 9.63] | 0.56 | .578 |
|  | HRMax | -4.39 | 3.83 | [-11.51, 2.73] | -1.15 | .257 |
|  | Debrief HRMax | -4.95 | 2.93 | [-10.39, 0.49] | -1.69 | .096 |
|  | Age | -3.96 | 3.18 | [-9.87, 1.94] | -1.25 | .217 |
|  | Female | 9.43 | 5.43 | [-0.66, 19.51] | 1.74 | .087 |
|  | Years of Experience | 1.98 | 3.30 | [-4.16, 8.11] | 0.60 | .551 |
|  | Lethal Force Error | -29.19 | 7.45 | [-43.03, -15.34] | -3.92 | >.001 |
|  | HR Rest | -0.17 | 2.65 | [-5.10, 4.76] | -0.07 | .948 |
| *Understanding* | Anticipation HRMax | 1.34 | 0.59 | [0.19, 2.49] | 2.28 | .023 |
|  | HRMax | -0.81 | 0.56 | [-1.90, 0.28] | -1.45 | .147 |
|  | Debrief HRMax | -0.49 | 0.42 | [-1.30, 0.33] | -1.18 | .239 |
|  | Age | -0.55 | 0.44 | [-1.40, 0.31] | -1.26 | .209 |
|  | Female | 2.24 | 0.92 | [0.44, 4.04] | 2.43 | .015 |
|  | Years of Experience | 0.34 | 0.41 | [-0.47, 1.15] | 0.83 | .408 |
|  | Lethal Force Error | -1.87 | 1.37 | [-4.57, 0.82] | -1.37 | 0.172 |
|  | HR Rest | .060 | 0.33 | [-0.59, 0.71] | 0.18 | .858 |
| *Lethal Force Error* | Cognitive Performance | -0.05 | 0.03 | [-0.12, 0.01] | -1.60 | .109 |
|  | Anticipation HRMax | 0.29 | 1.39 | [-2.44, 3.02] | 0.21 | .837 |
|  | HRMax | -3.01 | 1.47 | [-5.90, -0.13] | -2.05 | .040 |
|  | Debrief HRMax | 1.34 | 0.85 | [-0.32, 3.00] | 1.59 | .113 |
|  | HR Rest | 0.89 | 0.89 | [0.95, 2.65] | 1.00 | .317 |
|  |  |  |  |  |  |  |
